# Language of the Heart: Creating Digital Stories and Found Poetry to Understand Patients’ Experiences Living with Advanced Cancer

**DOI:** 10.3390/curroncol32020061

**Published:** 2025-01-23

**Authors:** Kathleen C. Sitter, Jessame Gamboa, Janet Margaret de Groot

**Affiliations:** 1 Faculty of Social Work, University of Calgary, Calgary, AB T2N 1N4, Canada; 2 Department of Psychiatry, Oncology, and Community Health Sciences, Cuming School of Medicine, University of Calgary, Calgary, AB T2N 2T9, Canada; jessame.gamboa@ucalgary.ca (J.G.); janet.degroot@albertahealthservices.ca (J.M.d.G.); 3 Department of Psychosocial Oncology and Rehabilitation Services, Holy Cross Site, 2202 2nd Street SW, Calgary, AB T2S 3C3, Canada

**Keywords:** advanced cancer, patient experiences, healthcare, digital storytelling, found poetry, poetic inquiry

## Abstract

In this article, we share our findings on patients’ experiences creating digital stories about living with advanced cancer, represented through found poetry. Over a period of 12 months, patients from the program “Managing Cancer and Living Meaningfully” (CALM) completed digital stories about their experiences living with cancer. Digital stories are short, personalized videos that combine photographs, imagery, narration, and music to communicate a personal experience about a topic of inquiry. Patient interviews were conducted about the digital storytelling process. Found poetry guided the analysis technique. It is a form of arts-based research that involves using words and phrases found in interview transcripts to create poems that represent research themes. This article begins with a brief overview of the psychosocial intervention CALM, arts in healthcare, and found poetry, followed by the project background. The found poems represent themes of emotional impact, legacy making, and support and collaboration. Findings also indicate the inherently relational aspect of digital storytelling as participants emphasized the integral role of the digital storytelling facilitator. What follows is a discussion on digital storytelling, which considers the role of found poetry in representing patient voices in the research process.

## 1. Introduction

In this article, we share our findings on patients’ experiences creating digital stories about living with advanced cancer, represented through found poetry. Over a period of 12 months, patients from the program “Managing Cancer and Living Meaningfully” (CALM) in a Western Canadian province completed individual digital stories about their experiences living with advanced cancer. Digital stories are short, personalized videos that combine photographs, imagery, narration, and music to communicate a personal experience about a topic of inquiry. Patient interviews were conducted about the digital storytelling process. Found poetry guided the analysis technique. It is a form of arts-based research that involves using words and phrases found in interview transcripts to create poems that represent research themes. Miller’s ICCEE approach to found poetry (*Immersion, Creation, Critical Reflection, Ethics, and Engagement*) guided the analysis technique, resulting in five found poems from the interview transcripts [1]. We begin with an overview of the psychosocial intervention CALM and the creative arts in healthcare with a focus on digital storytelling and poetry inquiry. What follows is the project background and the adapted method of Miller’s ICCEE. While CALM project details have been published elsewhere [2], in this article, we focus on patient experiences with digital storytelling based on the data analysis from the found poetry process. We conclude with a discussion on digital storytelling and patient narratives and consider the role of found poetry in representing patient voices.

### 1.1. Advanced Cancer and Psychosocial Interventions

Individuals with incurable cancer face numerous stressors, including heightened existential concerns and symptom burden due to the metastatic disease. Estimates of depressive symptom prevalence among individuals with advanced cancer affect at least 30% of individuals with advanced cancer [3]. Adverse consequences associated with depression among cancer participants include increased mortality rates, lower quality of life, higher symptom burden, and longer hospital stays [4,5,6,7,8]. Demoralization amongst patients with advanced cancer is also another consequence and is estimated to affect 25% of individuals with incurable cancer [9,10].

Research reveals that inter-professional palliative care that includes a psychosocial clinician is more likely to ensure psychological needs are met for patients with advanced cancer [11]. Additionally, when early integrated palliative care (i.e., at the time of diagnosis) addresses coping practices, there is a lower risk of depression and a greater quality of life [12,13,14]. Indeed, adopting a holistic approach to patient care with chronic illness has been well documented in the literature [15,16]. Seminal works such as Kleinman’s “The Illness narratives” focus on understanding illness through biological, cultural, and personal dimensions [16]. Kleinman’s work highlights the multifaceted nature of illness and the limitations of solely considering the biomedical model in interventions and patient outcomes, advocating for a holistic approach to patient care [16]. However, scholarship indicates that the implementation of psychosocial interventions early in the trajectory of palliative care is less systematically developed compared to physical symptom control or advanced care planning [12,17].

### 1.2. The Managing Cancer and Living Meaningfully (CALM) Intervention

The “Managing Cancer and Living Meaningfully” (CALM) psychological intervention was specifically created for patients with incurable solid tumour cancers [18]. The CALM intervention is a brief, semi-structured psychotherapeutic intervention for persons with advanced cancer who are invited to include an informal caregiver, e.g., spouse, adult son/daughter, or family member [18,19]. Practitioners meet patients as individuals with advanced illnesses facing mortality, acknowledging they are near death and have death anxiety. CALM therapy involves 3–6 individual sessions, approximately 45–60 min each, and is delivered over approximately 6 months. If necessary, additional sessions are offered. Throughout the sessions, four domains are covered: (1) symptom management and communication with healthcare providers; (2) changes in self and in relations with close others; (3) sense of meaning and purpose; and (4) sustaining hope, exploring the future and facing mortality. Although all domains are explored with each patient, the relative amount of time devoted to each domain varies according to their relevance to the patient [20]. Its efficacy has been well documented through psychotherapy evaluation studies [21,22]. CALM has been shown to have a greater effectiveness than usual care in reducing and preventing two important patient outcomes: depressive symptoms and preparation for the end of life [20,21].

### 1.3. Creative Arts and Healthcare

*“The richness of stories gives an understanding of the uniqueness of the patient’s fears and individual needs”*

[23] (p. 278).

In the practice of medicine, the creative arts can shape our understanding of illness and draw attention to aspects that might otherwise be overlooked [23]. Stories often include metaphoric images and provide a deeper understanding of individual needs while making sense of the experience. When individuals are unable to express themselves in language, there may be strength and validation found through art [23]. Imagination and creative engagement inherent in the arts can touch a deeper part of oneself and help connect with another.

In the healthcare setting, digital storytelling describes the practice of patients using digital media to tell their stories about a topic or issue from their own perspective. What distinguishes digital storytelling from other forms of storytelling is its multimodal approach. The process involves combining photographs, music, and personal narratives to create a short video about a personal experience that is written, narrated, and edited by the patient. The technique has gained significant momentum as a healthcare intervention strategy, particularly in the area of cancer [24,25,26,27]. Increasingly, digital storytelling is being applied in patient care research, as noted in a recent scoping review written by Park, Forhan, and Jones [28] and a systematic health review by West and colleagues [29]. The patient story or narrative of their illness is recognized as a research method itself [23]. Indeed, digital stories have gained significant focus as a method in health research to communicate first-person accounts of patient experiences in healthcare. Creative narratives near the end of life can become a significant act of engagement that creates a symbolic, emotional, and spiritual language about advancing illness and anticipation of death. Given the broad use of digital storytelling among people with cancer and carers, we were interested in learning about patient experiences of making digital stories about living with cancer, and we chose found poetry as a method of representation and analysis.

### 1.4. Found Poetry

In the last 20 years, there has been an interest in stretching the boundaries of modes of representation in research [30]. Arts-based representation mediates new forms of understanding, disrupting texts and bringing the researcher closer to the work [30]. Poetic inquiry is an arts-based research method that seeks to uncover diverse ways of understanding a topic [1]. There are multiple forms of poetic inquiry and various terminology describing this literary method. Prendergast’s meta-analytical study identified 230 studies of the use of poetry in social science qualitative research practices [31]. While she noted that participant-based studies generally draw on found poetry to represent data, the term poetic inquiry is an umbrella term covering multiple terminologies, including data poems [32], ethnopoetry [33], transcript poems [34], interview poems [35], poetic analysis [30], and research-generated poetry [36]. All of these examples are a form of “Vox participare—participant voiced poems” [31] that include research-written poems based on interview transcripts.

The tradition of found poetry is used to represent participant data [31]. Research-generated poetry is based on the words “found” in the research materials, i.e., transcripts (p. 990, [37]). Butler-Kisber defines found poetry as “taking the words of others and transforms them into poetic form” (p. 233, [30]). This approach to found poetry involves using interview transcripts where only the words of the participants are used to create a poetic rendition of a phenomenon [38]. As a form of found poetry, Glesne describes poetic transcription as “the creation of poemlike compositions from the words of the interviewees” (p. 202, [39]).

The potential power of found poetry is to do what poetry does: to synthesize an experience in a direct and effective way (p. 545, [31]). As an analytic tool within the inquiry process, it brings the researcher closer to the data that can yield new and important insights [30]. When we read found poetry, we are reminded of different ways in which we work with text when exploring multiple ways of understanding a topic [38]. In various analytic forms, researchers reorder and group texts from transcripts into identifying themes. In found poetry, the process serves to show rather than “tell”, as explained by Rath: “In crafting poems from the transcriptions of interviews, I do something with the data, rather than saying something about it” (p. 117, [36]). In this context, the use of found poetry serves as a valuable approach to challenge conventional ideas about what “constitutes accessible academic knowledge” (p. 123, [36]).

As an arts-based approach, found poetry leans toward the form and direction of poetry, but it does not strictly adhere to poetic conventions [39]. For instance, Glesne incorporates excerpts from interviews, creatively engaging with the words by drawing on traditional qualitative data analysis involving coding and organizing data by themes, then transitioning into the process of poetic transcription [39]. The process “approximates poetry through the concentrated language of the interviewee, shaped by researcher to give pleasure and truth. But the truth may be a ‘small t’ truth of description, re-presenting a perspective or experience of the interviewee, filtered through the researcher…the writing experiments with form in re-presenting research” (pp. 213–214, [39]).

Kendall and Murray also used poetic inquiry as an interpretive approach to interview data about patient experiences with lung cancer [33]. Kendall and Murray state that a clinician interpreting a patient’s history in poetic form may appreciate more of the emotional impact of the cancer journey [33]. As part of this research, the authors interviewed 20 patients and their caregivers [33]. They acknowledge that while this approach to analysis may make some researchers uncomfortable, all researchers make decisions that have implications for analysis and reporting [33].

The process of found poetry is both varied and emergent. Walsh created found poetry from interview data [37]. To create found poetry, the author would read and reread transcripts, make notes, remove words, and rework segments to further identify themes [37]. Walsh describes efforts to remain as faithful as possible to the original words while navigating decisions that were both artistic and academic: “I included only those phases that I saw as pertinent to the theme that I was emerging. I reordered phrases at times to improve clarity for the reader. I wrote, walked away, rewrote and revised in an ongoing recursive process….” (p. 990, [37]). Similar to Walsh’s approach to found poetry [37], we relied on transcripts from patient interviews to understand their experiences about creating digital stories about living with advanced cancer.

## 2. Materials and Methods

### 2.1. Project Background

Given the broad use of digital storytelling among people with cancer, we aimed to learn about patients’ experiences in creating digital stories about living with cancer. This was part of a mixed-methods study that assessed the feasibility of implementing CALM for patients with advanced cancer in a Western Canadian province alongside an established comprehensive psychosocial oncology program and a palliative care program. The study included two phases: the first involved evaluating referral patterns to CALM and the psychological benefits of CALM. A total of 69 individuals participated in phase one.

The second phase focused on the role of digital storytelling and involved two objectives: (1) to understand how digital storytelling may facilitate meaning-making related to the cancer experience and (2) to explore the participant experiences in creating their digital stories. A total of 24 patients from phase one consented to be contacted for phase two. From this group, six patients agreed to participate and completed a digital story. It is essential to highlight that we consider these numbers notably high, particularly given the context of working with patients at the end of their lives. Acknowledging the significant value of their time, we are deeply grateful for their generosity in choosing to spend these precious moments with us to create a digital story.

Each patient worked one-to-one with a team member trained in digital story facilitation. Individual digital story sessions ranged from one to two hours in length, with patients engaging in 3–6 sessions. (see Table 1). All sessions took place on Zoom, using the platform WeVideo. Once completed, each participant held an individual screening of their digital story with audience members chosen by the patient, which typically included family members, friends, the physician, the clinician who provided CALM for the patient, and team members from the CALM project. Audience numbers ranged from 5 to 60. Approximately one week after the screening, patients were interviewed about their experiences developing a digital story. A total of five interviews were completed (See Table 2). One participant was not interviewed about his experience, as he died shortly after finishing his story.

The interviews lasted 15 to 20 min and were audio recorded and transcribed. For this article, we focus on the second objective in phase two: to understand patient experiences in creating digital stories about living with cancer. As part of phase two, we engaged in found poetry as mode of data analysis using the transcripts from the patient interviews.

### 2.2. Analysis: Found Poetry

Found poetry is a form of research poetry and involves creating poems from words found in interview transcripts [1]. The process typically involves the researcher searching through the interview transcripts to identify keywords, phrases, or sentences based on the topic of inquiry and subsequently creating a poem or poem-like prose to arrange participants’ words into poetic form [1]. We chose to engage in found poetry as a means to achieve a deeper and more nuanced understanding of patients’ experiences in creating digital stories. As we were already working in the creative and multimodal space of visual storytelling, coupled with our research experiences in arts-based methods, we appreciate and understand how the arts open up diverse and immersive ways to explore a topic. Found poetry embodied a method that bridged the arts and science in representing patient voices in authentic and multifaced ways.

We drew on a critical medical humanities perspective to guide our process [1]. This perspective engages critical reflexivity, collaborative frameworks, power sharing, and self-empowerment [1]. Qualitative data from the transcripts were analyzed. Our process mirrored Miller’s approach to found poetry and hence was informed by her framework and adapted to meet our needs with two important distinctions [1]. First, as patients were in advanced stages of cancer, time was a critical consideration for engagement. Timing of sessions was often based on the availability and capacity of the patient. Second, where Miller drew on photovoice, we worked with digital storytelling. This difference is worth noting, as visual analysis of moving images is time-intensive. We made a conscious decision not to include imagery analysis with the interviews in the found poetry portion of our research for several reasons. First, the digital stories focused on individual experiences living with advanced cancer, which addressed the first objective, and we aimed to honour these narratives, which are first-person accounts carefully crafted with imagery, music, and voice, in their wholeness. Secondly, including the videos in our analysis would move us away from our question, which was focused on understanding the patient experience of creating a digital story.

#### Found Poetry: Our Process

In developing the poems, we were guided by Miller’s process, described through the metaphor and use of acronym ICCEE “Immersion, Creation, Critical Reflection, Ethics, and Engagement” [1]. With the beginning stage, *Immersion*, we engaged with the transcripts for long periods of time. As found poetry solely uses the participant’s words “found” in the transcripts, we highlighted words and sentences, culling words from the transcripts, noting memorable phrases, and identifying core themes. In developing the poems, we began with one of us refining the words from each transcript to create a poem. We then came together to read each poem aloud. We experimented with order and the location of the words on the page to consider the flow and feel. This is considered the 2nd step, *Creation.* Step 3 is *Criticality,* which refers to the quality of the poem. Miller cites Faulkner’s criteria of (i) artistic concentration, (ii) embodied experience, (iii) discovery/surprise, (iv) conditionality, (v) narrative truth, and (vi) transformation [1,40]. In our scheduled weekly meetings, we considered the second, third, and fourth steps when reviewing the poems, sharing our thoughts, and reworking the found poems based on our discussions. For instance, the poems included individual patient experiences, but through our discussions and interpretations of themes, we noticed several patients commented on a number of common experiences about digital storytelling, including the emotional impact of the experience, a form of legacy-making, and the support and collaboration they felt working with the primary digital story facilitator. In representing these experiences, these words and phrases were synthesized to create the found poems.

Step 4 of the ICCEE is *Ethics*. Miller states that where possible, researchers should share poems for viewing but adds an important caveat: “as researchers do not routinely return conventional qualitative data analysis to participants for approval, my pragmatic view is that poem member checking is ideal—but not compulsory” [1] (p. 36). While we agree that member checking is ideal, several participants died prior to this stage of completing the poems. Two patients provided appreciative feedback and made no edits to the found poems. Both expressed a sense of amazement at seeing their own words so eloquently reflected in poetic form. One patient was astonished to discover that the poem was composed entirely of their own words.

To ensure this study’s trustworthiness, we adhered to quality criteria that encompassed both member checking and reflexivity. This reflexivity involved critically reflecting on our questions and the diverse interactions we had with patients while maintaining an acute awareness of our own social locations and intersecting identities as healthy, well-educated women. This awareness underscored the importance of approaching the crafting of these poems with great care, recognizing them as a gift from patients who chose to share their precious time during the final stages of life. Aligning with Miller’s suggestion to include multiple excerpts of the raw data using the patient’s exact words [1], we engaged with the transcripts for extended periods of time and used only the words and phrases from the interview transcripts.

The final step, *Engagement*, calls for researchers to be familiar with how to write poetry, whether that is reading, practicing, or saying it out loud. To prepare, we read several books about poetic inquiry such as “Arts Based Research” by Tom Barone and Elliot Eisner [41], “Poetry as method: Reporting research through verse” by Sandra L. Faulkner [40], “Poetic inquiry II: Seeing, caring, understanding” by Kathleen T. Galvin and Monica Prendergast [42], and “Handbook of the Arts in Qualitative Research” by J. Gary Knowles and Ardra L. Cole [43]. We also read “The Sun and Her Flowers” by Rupi Kaur [44]. The books about poetry provided various examples of how to write poetry, considered words and order, and also included many examples.

## 3. Results

Through our analysis of the transcripts, five found poems were developed, and we identified three overarching themes in understanding patient experiences while creating digital stories about living with cancer: the emotional impact, legacy-making, and collaboration and support. Since the found poems intersect with the three core themes, each of the following sections begins with a brief introduction followed by one or two poems. The poems are intended to convey the essence of the themes, so we have deliberately limited additional commentary on their overall content.

### 3.1. Emotional Impact of the Digital Storytelling Process

Completing digital stories about experiences living with cancer held an emotional impact for patients. The process of selecting photographs, crafting and narrating their scripts, and choosing the music for their digital stories fostered layered reflection on the extensive impact cancer has had across so many aspects of their lives.
**It was perfect***It was perfect.**It was a great way to get my feelings across*
*To show people what I went through**To show myself what I went through*
*It’s nice to have that recording of your journey, going through life with cancer**To go through everything and piece it all together**It was very cathartic**It was kind of like writing your own obituary*
*I found it very helpful**People thought it was fabulous that I could do that**It was perfect for me.*

The poem unveils a fundamental value of the intervention: self-reflection. Notably, the patient likens this process to crafting one’s own obituary, hinting at contemplation of life and mortality amidst illness. The patient’s words underscore the importance of navigating the cancer journey while symbolizing a renewed outlook gained through the intervention.

### 3.2. Legacy and Meaning-Making

Another theme that emerged was legacy: patients described how creating their digital stories carried a profound sense of purpose in supporting others navigating advanced cancer. Through these digital stories, patients shared aspects of their personal journeys—encompassing diagnosis, treatment, care, and the vital support of family, friends, and healthcare teams. Reflecting on the process of creating these stories, patients expressed how their narratives might offer solace and connection to others facing similar challenges. In this way, their digital stories not only leave a meaningful legacy for their loved ones but also serve as a source of reflection for the broader community.
**It touches you**
*I truly believe in Storytelling**It touches you**It touches your soul**It touches your heart**It touches people**People understand storytelling**It’s an emphatic way of communicating**It brings positive energy and inspiration**It’s really great that I can do that**I’m really proud of it, what we have done**it helped me**Timelines depend on me, the patient**depends on my availability, the time he’s available**if he’s feeling good or not**It was a very positive experience**I want to share with other patients and caregivers**I want to help them find a way**I truly believe that it can help**it really adds value to people**It helps you mentally**It can help you**It can help other people understand what you’re going through the chemo, the radiation**what was going on then, where we are now**I would love to show it to the rest of the world.*

Digital storytelling dives into the depths of human experience, fostering introspection, self-discovery, and connections with oneself and others. It elicits a range of intense emotions, from fear and anxiety to warmth and hope, while nurturing resilience. The patient stresses its significance for raising awareness or empathy in others. The following poem also emphasizes the potential of digital stories in legacy storytelling.
**We did it***We did it**It wasn’t scary at all**It was very easy**easy to fit into schedule**short 5 sessions**just had to read the script**pick some pictures**the fun parts**it all went smoothly*
*we all have a story to tell**we all have a legacy to leave behind**I’m glad I had the opportunity to participate**It was very helpful*

Every individual carries a unique story and legacy, which can be powerfully conveyed through storytelling. Storytelling provides a meaningful way for individuals to find personal fulfillment while leaving a lasting impact on others. As a tool for legacy-building, storytelling allows individuals to share their experiences, impart wisdom, and spark inspiration. Furthermore, it has the potential to advocate for positive change in critical areas such as healthcare, research, and patient care, creating connections that resonate far beyond the individual narrative.

### 3.3. Support and Collaboration

The third theme centered on collaboration and support when creating digital stories. Patients emphasized the importance of the facilitator’s role, the connections and relationships formed, and the pace of the process, highlighting the significant impact these intersecting elements had on the patient experience while developing their digital stories about living with cancer.
**It was such a benefit***When you’ve got a terminal cancer, it’s so stressful**a hospital investing in something like this benefits so many people**i’m really grateful*
*It was really smooth**you knew me a little bit and what we’ve gone through**you handled that technology part**you assured me that you would be handling all of that**you worked your magic**i’m really grateful*
*As a team, we worked pretty good together**I didn’t feel that we emphasized enough of the self-soothing**you were just right on there so that it is more emphasized**i’m really grateful*
*It supported me**I learned a lot about myself**I learned a lot about my story**i’m really grateful*
*It supported my family**it got to include my husband, the family**i’m really grateful*
*It was such a benefit.*

Screening the stories with family members is also an important element in the digital storytelling process. Online screenings of digital stories facilitated further discussions with people central to the patients’ lives. While typically framed as a celebration of accomplishments, these screenings also functioned as legacy-making interventions. It allowed patients to reflect on their journeys with intention while receiving appreciation and gratitude from others. This process helped patients create meaningful “legacy” stories, providing a way to share their experiences with loved ones. Screenings lasted from 30 to 60 min, depending on how much people shared.
**More than Satisfied***You got to know who I am**We got to know each other*
*You picked out the most important pieces of my journey that would make the story**It’s important that the patients you speak to have to be open with you so that you can get the whole feeling of who they are**It’s important for patients to feel comfortable and trusting to make this the best story they can make it*
*You were just very very cooperative with me**Working with me for timing things, when we could do our interviews and when we couldn’t do them**You were patient and understanding*

*It was amazing, definitely more than satisfied with it.* The digital storytelling process is profoundly relational, where the facilitator’s role is integral to the development of the digital stories. It was clear through the interviews that patients felt their relationship with the facilitator was important to help patients create a digital story that authentically represents their cancer journey.

## 4. Discussion

Digital storytelling offers a powerful and multifaceted tool for patients with advanced cancer, allowing them to reflect on and share their experiences in ways that are deeply personal and meaningful. This project stands out for its focus on supporting individuals who might not otherwise engage in creative endeavors, highlighting the emotional and collaborative dimensions of the storytelling process. By centering patient needs and embracing flexibility, digital storytelling became a platform for legacy-making, emotional processing, and connection. The process facilitated meaningful reflections, fostered deeper insights, and provided a way for patients to share their journeys with loved ones, illustrating the significant impact of digital storytelling in advanced cancer care.

### The Role of Found Poetry

Found poetry, created from the words of participants, captures the essence of their stories in a condensed, evocative form. While poetry is used in healthcare research, its application in supporting patient understanding of their experiences engaging in digital storytelling is unique.

Found poetry reveals the personal significance of the digital storytelling process for individuals with advanced cancer. By centering the patients’ voices, this approach offers an intimate lens through which to explore the multifaceted and deeply meaningful process of crafting a digital story about living with cancer. The poetic structure allows for a more nuanced understanding of patient perspectives by providing a unique way to convey complex emotions and reflections that might be difficult to express through traditional narrative forms.

The creation of found poetry is, at its core, a deeply reflective process of engaging with data. By engaging in found poetry, we join others through a variety of experimental writing to explore the boundaries between science and art in representing research [39]. Immersing ourselves in the patient transcripts, we revisited patient’s words, striving to truly understand and honour their experiences. This process demanded an extraordinary level of care and attention as we worked to craft the poems. While we had employed various analytical approaches in previous research, this method felt profoundly different—more personal, more intimate. We were carefully amplifying the patients’ voices, seeking to represent them with authenticity and respect. The creative process opened a space for deeper exploration, allowing us to feel how themes flowed within through the transcripts. Every sentence, every phrase, and every form of presentation required—and received—our careful engagement, inviting us into a connection with the patients’ stories that was both meaningful and transformative.

## 5. Conclusions

This study demonstrates how digital storytelling and found poetry can provide meaningful ways to understand the experiences of patients. Found poetry, in particular, offered a thoughtful and creative approach to representing patient voices that might otherwise be difficult to articulate. This work highlights the importance of careful engagement with patient narratives and the value of creative approaches in enriching both healthcare research and understanding of patient experiences.

## Figures and Tables

**Table 1:** Digital story participants.

Age at First Session	Gender	Session #	Session Length in Minutes	Time to Complete Digital Stories	Digital Story Length	Digital Story Title
55	M	3	60–120	1 week	11:06	The Bridge
54	F	4	60	2 weeks	2:55	The transformation
59	M	6	60	6 months	5:57	No one can promise you tomorrow
70	F	4	60–120	3 weeks	6:57	Finding the light
55	F	3	60–120	3 weeks	4:42	Climbing my biggest mountain
62	F	4	60–120	4 weeks	4:52	Always been the caregiver

**Table 2:** Found poetry created from interview transcripts.

Digital Story Title	Completed Interview	Poem	Read the Poem
The Bridge	N	-	-
The transformation	Y	We did it	No
No one can promise you tomorrow	Y	It touches you	Yes
Finding the light	Y	It was such a benefit	No
Climbing my biggest mountain	Y	More than satisfied	No
Always been the caregiver	Y	It was perfect	Yes

## Data Availability

Data are unavailable due to privacy restrictions.
